# Dynamics of Fear‐Generalization Processes and Their Predictive Value for the Development of Anxiety‐Related Psychopathology in Adolescents and Adults: A Study Protocol for a Longitudinal Study

**DOI:** 10.1002/mpr.70094

**Published:** 2026-07-12

**Authors:** Jessica V. Reinhart, Katharina Hutterer, Heike Weber, Helena Strehl, Miriam A. Schiele, Katharina Domschke, Andreas Reif, Paul Pauli, Ulrike Lueken, Udo Dannlowski, Tina B. Lonsdorf, Bertram Müller‐Myhsok, Jürgen Deckert, Marcel Romanos, Julia Reinhard, Angelika Erhardt‐Lehmann, Andre Pittig

**Affiliations:** ^1^ Center of Mental Health Department of Child and Adolescent Psychiatry, Psychosomatics and Psychotherapy University Hospital of Würzburg Würzburg Germany; ^2^ Center of Mental Health Department of Psychiatry, Psychosomatics and Psychotherapy University Hospital of Würzburg Würzburg Germany; ^3^ Department of Psychiatry and Psychotherapy Faculty of Medicine Medical Center—University of Freiburg University of Freiburg Freiburg im Breisgau Germany; ^4^ Department of Psychiatry, Psychosomatic Medicine and Psychotherapy University Hospital Frankfurt Frankfurt Germany; ^5^ Institute of Psychology Chair of Psychology I University of Würzburg Würzburg Germany; ^6^ Department of Psychology Humboldt‐Universität zu Berlin Berlin Germany; ^7^ German Center for Mental Health (DZPG), Partner Site Berlin‐Potsdam Berlin Germany; ^8^ Institute for Translational Psychiatry University of Münster Münster Germany; ^9^ Department of Psychology Section for Biological Psychology and Cognitive Neuroscience University of Bielefeld Bielefeld Germany; ^10^ Institute of Systems Neuroscience University Medical Center Hamburg‐Eppendorf Hamburg Germany; ^11^ Max‐Planck‐Institute of Psychiatry Munich Germany; ^12^ Department of Clinical Epidemiology and Biometry University of Würzburg Würzburg Germany; ^13^ Department Genes and Environment Max‐Planck‐Institute of Psychiatry Munich Germany; ^14^ Translational Psychotherapy, Institute of Psychology University of Göttingen Göttingen Germany

**Keywords:** anxiety, avoidance, fear generalization, genetics, longitudinal

## Abstract

**Objectives:**

Anxiety disorders involve abnormal fear learning and exaggerated generalization, proposed as potential risk factors. However, it is unclear whether these processes reflect stable vulnerability markers or symptoms of psychopathology, as most research relies on cross‐sectional correlations and few longitudinal studies assess their predictive role in anxiety. Furthermore, little is known about the test‐retest reliability of fear learning paradigms, despite their suggested function as trait‐like markers for anxiety.

**Methods:**

This longitudinal study reassesses initially healthy adults after 3–12 years, and children first examined at ages 8–12, then 12–16, and now at 16–22. Participants repeat the “screaming lady” paradigm, including fear acquisition and generalization. We integrate fear learning, psychometrics, HPA‐axis activity, and (epi)genetics to assess the stability of fear generalization, its predictive value for later anxiety, and the link of fear learning to the HPA system.

**Conclusion:**

Our findings will clarify the long‐term stability of fear learning paradigms and evaluate their value as markers of anxiety risk. In addition, a random forest model will explore multiple risk factors and their interaction in anxiety symptomatology. This may improve understanding of anxiety disorders and support early detection and prevention.

**Trial Registration:**

Retrospectively registered on AsPredicted.org (#238,927)

## 1 Background

Anxiety disorders (ANX) affect up to 25% of the population (lifetime prevalence) and impose substantial personal and societal burden (Kessler et al. 2012). Most disorders emerge in early adulthood. However, disorder‐specific onset peaks have been observed in childhood, adolescence, and early adulthood, with changes in the nature of anxiety throughout development (Kessler et al. 2005; Solmi et al. 2022). ANX frequently co‐occur with other ANX or mental disorders (Hendriks et al. 2016). Therefore, identifying core etiological factors is crucial for early detection, prevention and treatment.

The transition from functional to pathological anxiety likely arises from complex interactions among (epi)genetic, environmental, fear learning factors. Altered fear learning, shaped by these influences, has been proposed as an endophenotype of pathological anxiety (Kausche et al. 2025). However, few studies integrate these factors to predict individual risk. This project addresses this gap by examining the temporal dynamics of (i) fear learning in relation to pathological anxiety, alongside (ii) environmental and (iii) molecular factors.

### 1.1 Fear Learning Processes

Pavlovian fear learning, comprising acquisition, generalization, and extinction, underlies both functional and pathological anxiety (e.g., Lonsdorf et al. 2017; Pittig et al. 2018, 2020) and has yielded powerful translational insights into ANX mechanisms and treatment (e.g., Hermans et al. 2006; Pittig et al. 2016). In acquisition, a neutral stimulus (NS) paired with an aversive unconditioned stimulus (US) becomes a conditioned fear stimulus (CS+) that elicits fear responses, while a safety stimulus (CS‐) is never paired with the US.

Individuals vary in fear acquisition (Lonsdorf and Merz 2017), but also in how strongly fear generalizes to stimuli resembling CS+ (generalization stimuli, GS). Although normally adaptive, excessive generalization to safe stimuli is a hallmark of anxiety (Dymond et al. 2015).

Early work linked ANX to heightened acquisition, but evidence is mixed. One meta‐analysis suggested increased fear in single‐cue paradigms (Lissek et al. 2005), whereas later ones pointed to elevated fear responding to CS‐, indicating impaired safety learning, and stronger affective responses to the CS+ (Duits et al. 2015; Kausche et al. 2025). For generalization, meta‐analyses show modest but consistent elevation in ANX compared to controls (Cooper et al. 2022; Fraunfelter et al. 2022). Anxious traits (e.g., trait anxiety, intolerance of uncertainty, neuroticism) are similarly linked to enhanced fear generalization in subclinical adults (Baumann et al. 2013, 2017; Sep et al. 2019), suggesting it may be a risk factor for pathological anxiety. However, these findings are correlational.

Prospective research on fear learning and later ANX is scarce, findings, especially on fear conditioning, remain mixed, and conclusions must be drawn with caution. Stronger fear generalization predicted higher anxiety symptoms after 6 months in students (Lenaert et al. 2014). Similarly, elevated threat expectancy for safety‐like stimuli predicted PTSD symptoms 2 years later beyond baseline (Lommen et al. 2024). Yet, a recent study found no link between generalization and distress over 6 months (Carpentier et al. 2024). Overall, evidence for fear generalization as a predictor of anxiety is inconclusive, with mixed findings likely influenced by developmental, environmental, or genetic factors.

### 1.2 Test‐Retest Reliability

Testing whether fear generalization alterations predispose to ANX assumes they are trait‐like, requiring test‐retest reliability (Torrents‐Rodas et al. 2014). For fear acquisition, evidence is mixed (for a summary see Klingelhöfer‐Jens et al. 2022, supplement): early work showed moderate‐to‐strong SCR reliability (Fredrikson et al. 1993), but recent studies report modest and measure‐dependent stability (Klingelhöfer‐Jens et al. 2022). For fear generalization, Torrents‐Rodas et al. (2014) calculated generalizability coefficients (G coefficients) to estimate the proportion of individual variation in responses to CSs and GSs, finding stable individual differences across 8 months in startle, SCRs, and threat expectancy (*R*_IRS_ = 0.17–0.34) (Torrents‐Rodas et al. 2014). SCRs showed fair reliability in fear acquisition and generalization, while threat expectancy was less consistent over 1–2 weeks (Cooper et al. 2023). Overall, fear generalization shows short‐ and mid‐term reliability, though subjective ratings appear less stable, and long‐term data are lacking.

### 1.3 Development

Fear learning is thought to develop non‐linearly with age, including stages of altered fear learning and memory (King et al. 2013). A meta‐analysis in youth with and without ANX found adult‐like patterns, showing elevated fear responses to both CS+ and CS‐ (Duits et al. 2015; Dvir et al. 2019).

Preliminary cross‐sectional research suggests vulnerable age periods and a non‐linear relationship between age and fear generalization: children (8–10) exhibit a U‐shaped pattern with heightened responses to both CS+ and CS‐, while adolescents (11–18) show a linear decline from CS+ through GSs to CS‐, similar to adults (Glenn et al. 2012). Consistently, previous findings from the present study's cohort indicated that children (8–10) displayed greater fear generalization than adults (Schiele, Reinhard, et al. 2016). Adolescents (13–18) showed reduced stimulus‐context discrimination compared to adults in a context‐dependent generalization task (Klein et al. 2021). In a MEG study, adolescents (14–17) showed higher US expectancy to a CS‐ than adults, suggesting that stimulus discrimination relies more on sensory than cognitive processes in this age group (Roesmann et al. 2022). Extending previous youth‐to‐adult comparisons, a study of children and adolescents (8–17) found that fear responses (arousal, threat expectancy, and skin conductance) declined with age across all stimulus types (Reinhard et al. 2022). Age‐related gains in stimulus discrimination were also linked to reduced overgeneralization in older participants.

Nonetheless, some evidence challenges excessive fear generalization as a risk factor for ANX. Adolescents with ANX (10–17) and controls showed similar generalization patterns, though patients had overall higher responses, suggesting magnitude rather than pattern differences as risk factor for the development of ANX (Reinhard et al. 2024).

### 1.4 Environmental Factors and Life Events

Environmental influences such as stressful life events, childhood trauma, and separation experiences were associated with higher risk for ANX (Klauke et al. 2010; Nugent et al. 2011). Stressful life events can disrupt multiple regulatory systems throughout development and, most likely if not exclusively, trigger the onset of clinical symptoms. Multiple studies showed that childhood and adolescence are particularly vulnerable phases, when stressors impact cortico‐amygdala function and fear‐ or anxiety‐related behavior (Ferrara et al. 2021; Vismara et al. 2020). Recent animal studies link raphe nuclei neurotransmitter shifts (glutamate to GABA) to fear generalization, a process facilitated by cortisol (Li et al. 2024). Childhood adversity increases the risk for adult affective symptoms, including ANX and depression (Grummitt et al. 2024), and predicts ANX onset, recurrence and reduced therapeutic response (Kuzminskaite et al. 2021). In fear learning, childhood adversity is linked to reduced stimulus discrimination due to blunted CS+ responding across acquisition and generalization (e.g., Klingelhöfer‐Jens et al. 2025). This pattern differs from that in ANX (Duits et al. 2015; Kausche et al. 2025), suggesting a distinct risk profile. Generalization patterns did not differ between those with and without childhood adversity (for review Ruge et al. 2024). Phenotypically, childhood trauma and adversity are associated with alterations in multiple systems, for example, dysfunction in the endocrine stress response mediated by the hypothalamic‐pituitary‐adrenocortical (HPA) system, maladaptive traits, accelerated epigenetic age, reduced prefrontal volume and increased amygdala activity (Zannas et al. 2018). Despite inconsistent evidence, most studies report mild HPA hyperactivity in adult ANX, for example, PD, GAD, and phobias versus depression (Erhardt et al. 2006; Juruena et al. 2020). A recent study linked HPA changes mainly to anxiety arousal, not fear or worry severity (Vinkers et al. 2021). In sum, these findings highlight how life events moderate the development of pathological anxiety.

### 1.5 Genetic and Epigenetic Factors

Genetic factors consistently contribute to the development of ANX (Ask et al. 2021; Strom et al. 2024), though its architecture is complex and heterogeneous. Developmental twin studies additionally showed genetic effects on anxiety traits in adolescence (Polderman et al. 2015), and prior work in our youth sample linked candidate genes to anxious personality and arousal ratings (Reinhard et al. 2020).

The genetic contribution to the etiology of ANX has been investigated through numerous linkage, candidate gene and genome‐wide association studies (GWAS) (for summary Ask et al. 2021). The latest GWAS in ANX identified over 51 replicated loci and implicated GABAergic signaling as a potential mechanism underlying genetic risk for ANX (Ask et al. 2021; Strom et al. 2024), enabling analyses of single novel genetic markers and cumulative genetic risk in fear‐related processes such as fear generalization. Polygenic risk scores (PRS) can be derived which summarize the contribution of all the disorder‐associated genetic variants for ANX into a single variable, enabling longitudinal studies to clarify the associated risk over time and disentangle genetic from environmental influences in ANX (Qi et al. 2024). Additionally, preliminary genome‐wide evidence shows that distress and fear are genetically distinct in ANX, supporting the distress and fear model in adults but having different structures in younger age groups (Waszczuk et al. 2014). Thus, genetic effects interact with environmental factors differently across ages, highlighting the need for study protocols that investigate genetic data from different ages within the typical ANX onset period.

Epigenetics refers to gene regulation without altering the DNA sequence. Such modifications can be time‐stable, heritable, and responsive to environmental influences. Epigenetic changes include histone modifications, small/micro‐RNA‐related gene silencing and DNA methylation (DNAm). DNAm research in ANX mostly focused on candidate gene studies in the past (Schiele and Domschke 2018), however, first epigenome‐wide (EWAS) case‐control and therapy‐related studies were completed (Czamara et al. 2022; Domschke et al. 2025; Iurato et al. 2017; Ziegler et al. 2019; for a review Schiele et al. 2020). DNAm, as an epigenetic process, has been repeatedly linked to stressful environmental influences (cf. Gottschalk et al. 2020) and subsequent gene‐regulatory changes with trajectory‐dependent sensitive windows (Zannas and Binder 2014). However, although distinct combinations of genes and environmental triggers act at sensitive developmental windows, others may operate continuously, underscoring the need for trajectory‐based investigations across life stages, as proposed here. Furthermore, evidence on how epigenetic markers potentially affect fear learning processes is still lacking, emphasizing the need to explore these molecular mechanisms in longitudinal studies.

### 1.6 Own Key Findings From the Previous Recruitment Waves and Aims of the Current Project

In summary, evidence linking fear learning to pathological anxiety is tentative and inconsistent, with most studies using cross‐sectional designs that cannot establish causality (Baumann et al. 2017). Prospective work is scarce, and fear generalization has only been studied over short intervals (2 weeks to 8 months), leaving longer‐term and developmental effects unclear. Life events and (epi)genetic factors are also associated with anxiety and may influence fear learning, but more longitudinal studies are needed to clarify their predictive role.

This project reassesses participants from two earlier funding periods of the Collaborative Research Centre TRR 58 “Fear, Anxiety, Anxiety Disorders” funded by the German Research Association, project Z02 (2013–2020), which recruited healthy volunteers at the Universities of Würzburg, Münster, and Hamburg to collect genetic, psychometric, and fear learning data. In Würzburg, 526 adults (mean age 26.6 (7.3), cf. Schiele, Herzog, Kollert, Schartner, et al. 2020; Schiele, Ziegler, et al. 2016; Stegmann et al. 2019) and 475 children (aged 8–12 years, mean age 9.7 (1.3), cf. Reinhard et al. 2020, 2024, 2022; Schiele, Reinhard, et al. 2016) were recruited in the 2nd funding period (2013–2016). Psychometric, life history, and (epi)genetic data were collected to investigate gene‐environment interactions, with (epi)genetic analyses focusing on candidate genes (cf. Gottschalk et al. 2019; Notzon et al. 2016; Reif et al. 2014; Schartner et al. 2017; Schiele et al. 2020; Schiele et al. 2016) and complemented by GWAS/EWAS approaches (cf. Deckert et al. 2017; Domschke et al. 2025). In the 3rd recruitment period (2016–2020), 485 more adults (mean age 24.2 (5.4)) (cf. Schiele, Herzog, Kollert, Bohnlein, et al. 2020; Schiele, Herzog, Kollert, Schartner, et al. 2020) were added, and 337 children (mean age 12.3 (4.0)) from the earlier cohort were re‐assessed (> 70% retention) (Reinhart et al. Forthcoming). Overall, 1011 adults and 475 children completed prior assessments at the Würzburg site; 788 adults and 337 children are now eligible (see methods) for follow‐up.

This study thus offers a unique opportunity to examine time‐dependent changes in anxiety‐related endophenotypes in well‐characterized initially healthy cohorts. The main goal is to assess how fear generalization processes relate to anxiety symptomatology over time, and how genetic and environmental factors moderate this relationship. Specifically, the project investigates the temporal malleability of fear generalization, its predictive value for psychopathology across the age spectrum, and the integration of molecular and environmental data to better understand anxiety symptomatology. The key research objectives are as follows:

1. *Assess the stability in fear acquisition and generalization processes:* This will involve replicating findings from Cooper et al. (2023) and incorporating fear acquisition and generalization indices proposed by Stegmann et al. (2019).
2. *Prediction of the development of anxiety‐related psychopathology:* This aim focuses on evaluating the predictive value of fear acquisition, generalization, alongside comprehensive psychometric data, life events, (epi)genetic factors, and stress reactivity.
3. *Evaluate the relationship of fear acquisition and generalization processes and HPA axis activity:* The study will analyze how these processes relate to markers of HPA axis activity, such as cortisol awakening response (CAR).

## 2 Methods

### 2.1 Description of Previous Assessment Waves

**FIGURE 1:**
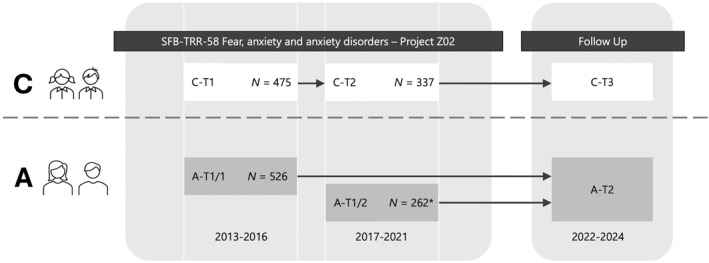
Recruitment plan. The follow‐up study involves the recruitment of participants of the child and adolescent sample who previously participated in both prior assessments. Additionally, all T1 adult participants who took part in the fear generalization paradigm will be recruited. In total, we plan to contact *N* = 337 children from C‐T2, *N* = 526 adults from A‐T1/1, and *N* = 262 adults A‐T1/2 (*total A‐T1/2: *N* = 485, only a subgroup participated in the fear learning paradigm). We anticipate a re‐recruitment rate of 50% for adolescents and 40% for adults. C = Child and adolescent cohort. A = Adult cohort.

Children and adolescents were initially recruited from primary and secondary schools in the greater Würzburg area as part of SFB‐TRR‐58 subproject Z02. Adult participants were recruited from the community and later through an online platform. In cohort A‐T1/2 (see Figure 1), only a subsample of the adult cohort participated in the fear learning paradigm. The study was approved by the ethical committee of the medical board of the university of Würzburg (256/21).

### 2.2 Recruitment

In this study, all Würzburg adults from two prior Z02 cohorts who completed the fear learning paradigm, as well as children and adolescents who participated in both earlier assessments and consented to recontact, are invited. This yields two timepoints for adults (T1 > T2) and three for youths (T1, T2 > T3; see Figure 1). Earlier assessments included extensive psychometric evaluations of anxiety symptoms, a fear conditioning and generalization paradigm, and genetic phenotyping.

Re‐recruitment is expected at ≥ 40% for adults and ≥ 50% for adolescents. Exclusion criteria include acute psychosis, suicidality, pregnancy, and illicit drug use (verified by urine toxicology). Unlike initial enrollments (Table 1), participants with psychopathologic traits are intentionally included to track longitudinal development.

**TABLE 1:** Overview and eligibility criteria of the initial and follow up sample.

	A‐T1/1 and A‐T1/2	C‐T1 and C‐T2	Follow‐up
Inclusion criteria	Age between 18 and 50 years Caucasian background Right‐handedness Fluency in German	Age between — 8 and 12 years (C‐T1) or — 12 and 16 years (C‐T2) Caucasian background Right‐handedness Fluency in German	Participants of adult cohort A‐T1/1 and A‐T1/2 that performed the paradigm Participants of the child and adolescent cohort C‐T1 and C‐T2 that took part in both assessments
Exclusion criteria	Major clinical disorders Pregnancy Severe medical conditions Ongoing medication intake Use of illegal drugs as assessed by urine toxicology More than 14 glasses alcohol per week More than 20 cigarettes per day More than 4 cups caffeine per day	Severe somatic conditions Intake of psychoactive medication Low intelligence (IQ < 85)	Pregnancy Use of illegal drugs as assessed by urine toxicology

Participants are first contacted in writing (email or letter), followed by a telephone call to provide more details and confirm eligibility. Oral informed consent is obtained, and interested participants are invited to complete an additional CAR measurement. For those unable to visit Würzburg, online options are available for clinical interviews and psychometric assessments.

### 2.3 Current Procedure

**FIGURE 2:**
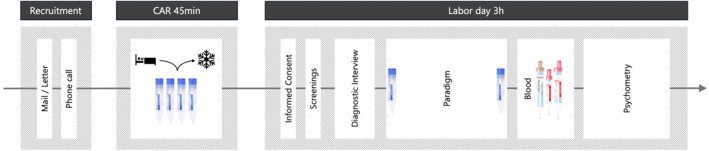
Schematic representation of study procedure. CAR = Cortisol Awakening Response.

All participants provide written informed consent. Prior to the experimental procedure, in a subgroup of participants samples for CAR analysis are collected following recommendations in Adam and Kumari (2009). Furthermore, all participants undergo urine testing to screen for substance use and pregnancy, followed by a diagnostic interview. Saliva samples for cortisol analysis are taken immediately before and after the fear acquisition and generalization paradigm, the study's core assessment, and blood samples are collected afterward. Participants then complete a comprehensive psychometric evaluation (see Figure 2 for an overview of the procedure). Remuneration is €50 for in‐person participation and €20 for online participation.

### 2.4 Screenings

Participants are screened for substance use and pregnancy before the experiment. Substance use is assessed with the Surestep Drug Test Dip Card, a multi‐panel immunoassay detecting various drugs and their metabolites in urine, while pregancy is checked using the Cleartest hCG Professional Pregnancy Test, a reliable and sensitive immunoassay for human chorionic gonadotropin (hCG) in urine. If either test returns a positive result, the assessment will be discontinued. All tests are administered following the manufacturers' guidelines to ensure accuracy and reliability.

### 2.5 Diagnostic Interview

The Mini‐DIPS (Diagnostic Interview for Mental Disorders, Margraf and Cwik 2017) is a semi‐structured interview conducted by a trained clinical psychologist. It is a widely used, standardized assessment of anxiety and other mental disorders, based on DSM‐5 and ICD‐10 criteria, and validated for individuals aged 16 and above. Interviews typically last 30–90 min, depending on symptom complexity. For participants unable to attend in person, the interview is conducted remotely via video conferencing.

### 2.6 Psychometry

To analyze the trajectory of anxiety symptomatology and its interaction with environmental factors, we employ an assessment battery based on measures from previous assessments (see Table 2 for details). We deliberately modified the battery to prioritize instruments that best capture psychopathology at follow‐up for predictive purposes, rather than adhering strictly to continuity with prior assessments.

**TABLE 2:** Overview of psychometrics across assessments.

Construct	Instrument	A‐T1/1	A‐T1/2	C‐T1	C‐T2	Follow‐up
Sociodemographic, health behavior	General questionnaire	X	X	X (P)	X (P)	X
Psychopathology	MINI	X	X			
	Kinder‐DIPS			X (C + P)	X (C + P)	
	Mini‐DIPS					X
	CGI					X
Intelligence	CFT‐20 R			X (C)	X (C)	
General anxiety symptomatology	STAI‐T	X	X			
	STAI‐C			X (C)	X (C)	
	BAI	X	X			X
	DSM‐5 dimensional anxiety scales		X			
	PROMIS anxiety					X
Anxiety sensitivity	ASI	X	X			
	CASI			X (C)	X (C)	
	ASI‐3	X	X			X
Panic/agoraphobia	ACQ	X	X			
	PAS					X
Social anxiety	LSAS	X	X			
	SPAI	X	X			X
	SPAIK			X (C)	X (C)	
Generalized anxiety	PSWQ	X	X			X
Phobias	FAS	X	X			
	PHOKI			X (C)	X (C)	
	ASA‐27		X			
	TAI			X (C + P)	X (C + P)	
General psychopathology	PROMIS‐29 profile (modified)					X
	BSI‐18					X
	YSR (screening)			X (C)	X (C)	
	CBCL (screening)			X (P)	X (P)	
Depression	ADS‐K	X	X			
	BDI‐II		X			X
	DIKJ			X (C)	X (C)	
Emotion regulation	CERQ short	X	X			
Affectivity	PANAS trait	X	X			
Intolerance of uncertainty	UGTS					X
Rejection sensitivity	RSQ‐9					X
Social desirability	SDS‐CM	X	X			
Temperament	TEMPS		X			
Behavioral approach	BIS/BAS	X	X			
Coping	Brief‐COPE		X			
	GSE	X	X			
Stress	TICS		X			X
	KFB		X			
	SVF‐78		X			X
	SVF‐K			X (C)	X (C)	
Social support	BSSS		X			X
	SS‐A		X			
Resilience	KIDSCREEN‐27			X (C + P)	X (C + P)	
	SDQ			X (C + P)	X (C + P)	
Parental behavior	ESF			X (P)	X (P)	
	PBQ‐AC‐M			X (P)	X (P)	
Parents' psychopathology	BAI			X (P)	X (P)	
	BDI‐II			X (P)	X (P)	
	SCL‐90‐R			X (P)	X (P)	
Life events	Life calendar	X	X (mod.)			
	LTE	X	X (mod.)			
	ZLEL			X (C)	X (C)	
	Munichs event list					X (mod.)
Childhood traumata	CTQ	X	X			
	CTS					X

*Note:* (C) = Self‐report by children and adolescents; (P) = Parent report.

Abbreviations: ACQ, Agoraphobic Cognitions Questionnaire (Chambless et al. 1984); ADS‐K, Allgemeine Depressionsskala Kurzform (Hautzinger 2012); ASA‐27, Adult Separation Anxiety Questionnaire (Manicavasagar et al. 2003); ASI, Anxiety Sensitivity Inventory (Reiss et al. 1986); ASI‐3, Anxiety Sensitivity Index‐3 (Taylor et al. 2007); BAI, Beck Anxiety Inventory (Beck et al. 1988; Brown and Steer 1988); BDI‐II, Beck Depression Inventory‐II (Beck et al. 1996); BIS/BAS, Behavioural Inhibition and Activation Scale (Carver and White 1994); Brief‐COPE, Brief Coping Orientation to Problems Experienced Inventory (Carver 1997); BSI‐18, Brief Symptom Inventory‐18 (Derogatis and Fitzpatrick 2004); BSSS, Berlin Social Support Scales (Schwarzer and Schulz 2003); CASI, Anxiety Sensitivity Index for Children (Schneider et al. 2009); CBCL (Screening), Child Behaviour Check List (Achenbach and Edelbrock 1991a); CERQ, Cognitive Emotion Regulation Questionnaire (Garnefski et al. 2001); CES‐D, Center of Epidemiologic Studies—Depression scale (Radloff 1977); CGI, Clinical Global Impression Scale (Guy 1976), CFT‐20 R, Grundintelligenztest Skala 2: Revision (Weiß 2006); CTQ, Childhood Trauma Questionnaire (Bernstein 1998); CTS, Childhood Trauma Screener (Grabe et al. 2012); DIKJ, Depressions‐Inventar für Kinder‐und Jugendliche (Stiensmeier‐Pelster et al. 1989); DSM‐5 Dimensional anxiety scales (Lebeau et al. 2012); ESF, Elternstressfragebogen (Tischler and Petermann 2015); FAS, Fragebogen zur Angst vor Spinnen (Rinck et al. 2003); GSE, Generalized Self‐Efficacy Scale (Schwarzer 1995); KIDSCREEN (Ravens‐Sieberer et al. 2001); Kinder‐DIPS, Diagnostisches Interview bei psychischen Störungen im Kindes‐und Jugendalter (Schneider et al. 2017); Life Calendar (Caspi et al. 2003); LSAS, Liebowitz Social Anxiety Scale (Liebowitz 1987); LTE‐Q, List of Threatening Experiences (Brugha and Cragg 1990); MINI, The Mini‐International Neuropsychiatric Interview (Sheehan et al. 1998); Mini‐DIPS, Diagnostisches Kurz‐Interview bei psychischen Störungen (Margraf and Cwik 2017); Munichs event list (Maier‐Diewald 1983); PANAS, Positive and Negative Affect Schedule (Watson et al. 1988); PAS, Panic and Agoraphobia Scale (Bandelow 1999); PBQ‐AC‐M, Parent Beliefs Questionnaire (Nauta et al. 2002); PHOKI, Phobiefragebogen für Kinder und Jugendliche (Döpfner 2006); PROMIS Anxiety, Patient‐Reported Outcomes Measurement Information System (Cella et al. 2007); PROMIS‐29 Profile, Patient‐Reported Outcomes Measurement Information System (Cella et al. 2007); PSWQ, Penn State Worry Questionnaire (Meyer et al. 1990); RSQ‐9, Rejection Sensitivity Questionnaire‐9 (Downey and Feldman 1996); SCL‐90‐R, Symptom Check List (Derogatis and Fitzpatrick 2004); SDQ, Strengths and Difficulties Questionnaire (Goodman 1997); SDS‐CM, Social Desirability Scale (Crowne and Marlowe 1960); SPAI, Social Phobia and Anxiety Inventory (Turner et al. 1989); SPAIK, Soziale Phobie‐und Angstinventar für Kinder (Melfsen et al. 2001); SS‐A, Social Support Appraisals Scale (Vaux 1988); STAI‐C, Spielberger Trait Anxiety Inventory for Children (Schmitz et al. 2011); STAI‐T, Spielberger Trait Anxiety Inventory; SVF, Stressverarbeitungsfragebogen (Janke and Erdmann 2002); TAI, Test Anxiety Inventory (Spielberger and Gonzalez 1980); TEMPS‐A, Temperament Evaluation of Memphis, Pisa, Paris and San Diego‐autoquestionnaire (Akiskal et al. 2005); TICS, Trier Inventory for Chronic Stress (Schulz et al. 2004; Becker 2004); UGTS, Ungewissheitstoleranzskala (Dalbert 1999); YSR (Screening), Youth self‐report and profile (Achenbach and Edelbrock 1991b); ZLEL, Züricher Lebensereignisliste (Steinhausen and Winkler Metzke 2001).

### 2.7 Fear Acquisition and Generalization Paradigm

**FIGURE 3:**
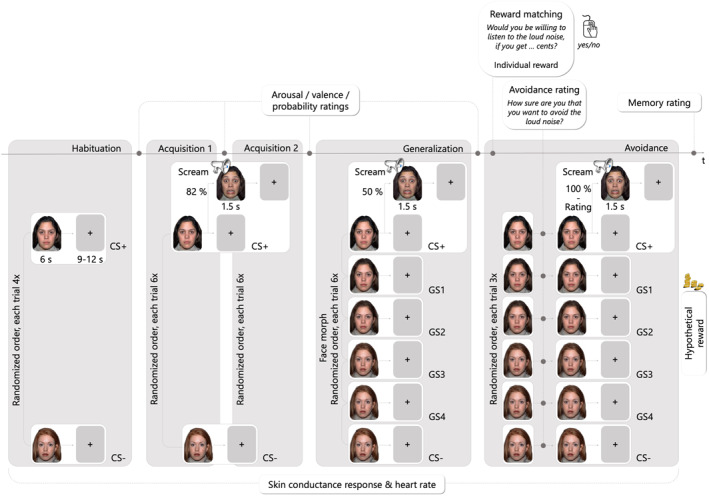
Overview of the paradigm, including fear conditioning, fear generalization, and costly avoidance.

Participants undergo the fear acquisition and generalization paradigm used in earlier assessments (Schiele, Reinhard, et al. 2016), using neutral female faces as conditioned stimuli (03F_NE_C, 10F_NE_C, NimStim Face Stimulus Set; Tottenham et al. 2009). CS+ is paired with a fearful face and aversive scream (International Affective Digitized Sounds, IADS, FemScream2, No. 276; Bradley and Lang 1999), while CS‐ remains unpaired. The experiment includes three phases: pre‐acquisition (unreinforced CS+ and CS‐), acquisition (CS+ reinforced on most trials, while CS‐ remains unpaired), and generalization (CS+, CS‐, and four morphed generalization stimuli, GS1–GS4, with partial CS+ reinforcement). Stimuli are presented with randomized order and intertrial intervals. After each phase, participants rate stimuli on arousal, valence, and US expectancy. An overview of the fear acquisition and generalization paradigm is shown in Figure 3. Compared to earlier assessments, fewer generalization trials are included (see Table 3), with analyses focusing on this first block.

**TABLE 3:** Specification of experimental phases in the fear conditioning paradigm.

Phase	A‐T1/1, A‐T1/2, C‐T1, C‐T2	Follow‐up
Pre‐acquisition	8 trials 4 CS+ (no US) 4 CS‐	8 trials 4 CS+ (no US) 4 CS‐
Acquisition block 1	12 trials 6 CS+ (of which 5 US at 95 dB) 6 CS‐	12 trials 6 CS+ (of which 5 US at 95 dB) 6 CS‐
Acquisition block 2	12 trials 6 CS+ (of which 5 US at 95 dB) 6 CS‐	12 trials 6 CS+ (of which 5 US at 95 dB) 6 CS‐
Generalization block 1	36 trials 6 CS+ (of which 3 US at 95 dB) 6 CS‐ 6 GS1, 12 GS2, 12 GS3, 12 GS4	36 trials 6 CS+ (of which 3 US at 95 dB) 6 CS‐ 6 GS1, 6 GS2, 6 GS3, 6 GS4
De‐generalization	*only for A‐T1/2 and C‐T2* 50 trials in two blocks In detail see [125]	—
Generalization block 2	36 trials 6 CS+ (of which 3 US at 95 dB) 6 CS‐ 6 GS1, 12 GS2, 12 GS3, 12 GS4	—
Extinction	*for C‐T1, optional for C‐T2* 18 CS+ 18 CS‐ without US	—
Avoidance	—	18 trials 3 CS+ (of which ≤ 3 US at 95 dB) 3 CS‐ 3 GS1, 3 GS2, 3 GS3, 3 GS4

*Note:* Italicized text indicates phases that were administered only to the specified cohorts at the respective assessment timepoints.

#### 2.7.1 Avoidance

After the generalization paradigm, participants complete a costly avoidance task to dimensionally measure safety behavior, adapted from Wong and Pittig (2022). A reward threshold for hearing the scream is first determined with yes/no monetary offers. Based on this threshold, participants rate their willingness to avoid the scream (0%–100%) for each CS and GS, where higher avoidance decreases both scream probability and the potential reward. After each rating, US expectancy is assessed. Importantly, CS‐ and GS trials are never reinforced, and all rewards are hypothetical, which effectively modulates avoidance behavior (Pittig and Dehler 2019; Pittig and Scherbaum 2020). Finally, participants identify which image was paired with the scream in prior assessments and rate their confidence to control for memory effects. The whole experiment lasts approximately 45 min.

#### 2.7.2 Physiological Recordings and Data Reduction

Heart rate and skin conductance responses are recorded at 1000 Hz using Brainproducts hardware and software. Heart rate variability (HRV) is assessed before and after the paradigm via ECG electrodes, while participants watch a neutral video. RR intervals are processed and analyzed in Kubios HRV software to derive markers for autonomic nervous system activity. Skin conductance response (SCR) is measured on the left hand, range‐corrected, log‐transformed, and averaged per stimulus, with participants showing mean responses < 0.02 μS classified as non‐responders and excluded.

### 2.8 Bioprobes

#### 2.8.1 Blood Cell Analysis

Blood samples are collected after the experimental paradigm to analyze blood cell composition and routine indices. The total blood volume collected is 18.1 mL, divided as follows: 9 mL EDTA for (epi)genetics and blood cell counts, 7.5 mL serum for metabolomics and hormone analyses, and 1.6 mL EDTA specifically for a differential blood count. Serum is centrifuged 20 min after collection at 2500g for 15 min at 4°C. Samples are stored at −80°C for later analysis. All participants had previously been genotyped at the genome‐wide association study (GWAS) level using high‐throughput genotyping arrays (1st wave PsychChip and 2nd wave GSA 3.0).

#### 2.8.2 HPA‐System

On the endocrine level, cortisol is measured from four saliva samples collected at home in the morning after awakening, using a provided test kit and instructions. Ideally, sampling occurs on the study day or a typical working day close to it: the first sample at awakening, followed by three more at 15‐min intervals over 45 min. Participants are asked to avoid food, drink, or physical activity, and are encouraged to remain in bed engaging only in low‐arousal activities such as reading. After collection, participants store the samples in a freezer until bringing them to the study site. If needed, the study team arranges sample pickup within the Würzburg area (for recommendations see Stalder et al. 2016, 2022). Additionally, two saliva cortisol samples are collected pre‐ and post‐fear generalization to assess task‐related stress responses.

#### 2.8.3 (Epi)Genetics

Genetic analyses on dimensional and categorical levels will focus on current anxiety phenotypes and changes since the initial assessment, evaluating how polygenic risk scores influence the development of fear generalization and anxiety symptoms over time. Analyses will also consider top candidate gene variants from recent GWAS in ANX (ANGST, UKBiobank, iPsych, PGC, such as *TMEM132D, GLRB*, *PDE4B, CAMKMT, RBFOX1, NTRK2, TMEM106, MYH15, SATB1and ESR1*) and replicated (epi)genetic risk variants for ANX from earlier recruitment waves (*NPSR1, CRH, CRHR1, ADCYP, ADCYAP1, SLC6A4, FAAH, MAOA, COMT, HTR1A, OXTR*). Epigenetic analyses will determine DNAm of specific CpGs in candidate genes listed above and conduct epigenome‐wide analyses using Infinium Methylation Bead Chips (> 935,000 CpGs across the genome). All analysis will be corrected for age, sex, smoking and body mass index as important determinants of DNAm.

An overview of bio‐probe analyses across assessment waves is provided in Table 4.

**TABLE 4:** Overview of bio‐probe analyses across assessments.

	A‐T1/1, C‐T1	A‐T1/2, C‐T2	Follow‐up
Sample	2*10 mL EDTA	2*10 mL EDTA	9 mL EDTA 1.6 mL EDTA 7.5 mL serum
A priori genotyping	X	X	X
Post‐hoc genotyping	X	X	X
Whole genome sequencing (WGS)	X	X	X
Epigenetic analyses (DNAm)			X
Gene × environment analyses			
Gene × environment × coping analyses	X	X	X
Blood cell analysis		X	X
Polygenic risk scores		X	X
Neuroimaging backbone module		X	
HPA‐system			X

*Note: A priori genotyping:* Probability of selected genes being involved in anxiety endophenotypes, assessed using rapid low‐throughput and high‐throughput genotyping methods. *Post‐hoc genotyping:* Validation of genetic variants based on prior hypotheses (e.g., animal studies or prior research findings). *Whole Genome Sequencing (WGS):* Comprehensive analysis of all genetic variations. *Genome‐Wide Association Studies (GWAS):* Broad screening for variant‐trait associations. *Gene × environment (× coping) analyses:* Stepwise multivariate regression analyses linking genetics, environment, and coping, extended by epigenetic data (e.g., DNAm). *Blood cell analysis:* Blood composition (e.g., red cell distribution width) and routine indices as indicators of psychopathology. *Polygenic risk scores:* Analysis to estimate the cumulative genetic risk for anxiety‐related traits. *Neuroimaging backbone module:* Brain morphology (structural MRI), functional connectivity (resting‐state fMRI), and data analysis at categorical (anxiety disorders), dimensional (anxiety sensitivity), and epigenetic levels. *HPA‐System:* Salivary cortisol measurements (e.g., after awakening and in response to a fear generalization paradigm) to evaluate stress responses.

### 2.9 Statistics

Main analyses address our three major research questions: (i) stability of fear acquisition and generalization processes, (ii) prediction of the developed anxiety‐related psychopathology, and (iii) the relationship of fear acquisition and generalization processes and saliva cortisol levels. Analyses may be adjusted for new methods or data quality (e.g., missing data, distributions).

#### 2.9.1 Stability of Fear Generalization Processes

To examine the stability of fear generalization, we will calculate established indicators and firstly adapt the approach outlined by Cooper et al. (2023). Linear mixed models with a fixed effect of stimulus for the acquisition phase and generalization will test differential fear conditioning. Furthermore, building on the approach by Cooper et al. (2023), additional models will be applied to examine mean‐level changes in fear responses over time. These models will include a random intercept for participants and random slopes for timepoint, as well as fixed effects of stimulus, phase (acquisition only), timepoint (T1, [T2,] FU), and their interactions. To investigate individual patterns of fear generalization, additional mixed‐effects models will be fitted to examine the effect of timepoint on the Linear Deviation Score (LDS). Age and the Age × Timepoint interaction will be included in all models to account for variability in the interval between the initial assessment and follow‐up, as well as cohort‐related age differences. As age effects are not central to the present research question, they will not be considered further. A more fine‐grained modeling of temporal variability will be implemented in the prediction analyses (see below *Prediction of anxiety symptomatology*).

Next, we aim to replicate the variance component analysis based on the principles of generalization theory to examine how variance is distributed across different levels of the model and to evaluate the relative contributions of individual differences, stimuli, and sessions to the observed effects (Cooper et al. 2023).

Further, we will assess intraindividual stability (i.e., within‐subject stability of response patterns) and change (i.e., within‐subject reliability of change) using the coefficients *R*_IRS_ and *R*_C_. *R*_IRS_, as used by Torrents‐Rodas et al. (2014), quantifies the proportion of within‐person variance in stimuli responses that remains stable across time. This measure is well‐suited to capture stability of generalization patterns, as it isolates the consistency of individual differences in response patterns over repeated sessions. To complement this, we will also calculate the *R*_C_ coefficient (Cooper et al. 2023), which measures the reliability of change in responses across individuals between timepoints. It quantifies how much non‐stable variance in responses reflects changes over time. Larger *R*_C_ suggest reliable tracking of systematic changes in response patterns across sessions.

We will also calculate Intraclass Correlation Coefficients (ICCs) to assess response consistency. Beyond Cooper et al. (2023)'s stimulus‐specific ICCs for acquisition and generalization phases, we will include three additional indices (Stegmann et al. 2019): ICCs based on the Linear Deviation Score, the Mean Response across all stimuli, and CS‐Differentiation, enabling interpretation of individual response patterns during generalization.

To investigate age‐related trajectories of fear generalization, we will apply likelihood‐based linear mixed models in the combined child, adolescent and adult samples depending on resulted estimates (Mallinckrod et al. 2008). This approach allows joint analysis of all timepoints based on a multivariate model useful to analyse longitudinal continuous outcomes.

#### 2.9.2 Prediction of Anxiety Symptomatology

For exploratory prediction analysis, we will compute an *Anxiety Factor Score*, yielding a single, streamlined, and reliable index of anxiety‐related psychopathology, based on Pittig et al. (2023), but using follow‐up‐based standardization. Each psychometric measure will be z‐standardized using the mean and standard deviation of the full sample at follow‐up and then averaged across anxiety‐related measures (i.e., BAI, PROMIS Anxiety, ASI‐3, PAS, SPAI, and PSWQ). Importantly, this composite reflects relative anxiety severity at follow‐up rather than intraindividual change over time.

To account for baseline differences in symptom levels, baseline anxiety will be included as a covariate in all longitudinal prediction models, thereby allowing the examination of predictors of follow‐up anxiety above and beyond initial symptom levels. Baseline anxiety will be operationalized as a composite score of anxiety symptomatology derived from the measures available at baseline, following the same general procedure as described above.

Next, linear mixed models expand the model of anxiety prediction and fear generalization with polygenic risk scores derived from publicly available Anxiety GWAS (Strom et al. 2024), cortisol awakening response (area under the curve of cortisol), early trauma and life events to explore each variable's contribution to anxiety. As described above, variability between time points will be included by modelling age as a quantitative variable and allowing for corresponding interactions with measurement timepoints. To further model this, we will also account for time‐warping in these analyses along the lines of the fdasrvf package in R (https://cran.r‐project.org/package=fdasrvf).

Moreover, random forest methods will integrate complex variables (e.g., GWAS and DNAm), combining multiple decision trees to obtain stable, accurate predictions via cross‐validation. Each tree votes, and the class with most votes determines the final classification. As predictors we will include fear generalization indices, psychometrics, life events, endocrine and molecular measures. Random forests resist overfitting, particularly under cross‐validation and with good predictive power. Besides, they handle missing data effectively, preserving predictive power.

For both the random forest and linear mixed models, we will use repeated nested cross‐validation to get a reliable estimate of generalization error expected in an unseen sample (see e.g. and following the ideas of Rost et al. 2022). In addition, we will perform permutation analysis encapsulating the complete prediction model building to investigate and confirm that the model building process as outlined per se will not lead to overfitting, meaning that the distribution of the performance metrics do indeed follow expectations under the null.

#### 2.9.3 Stress Reactivity

To examine links between fear generalization and HPA activity, we will correlate CAR, quantified as area under the curve with respect to the increase (AUCi), which reflects HPA‐axis stress reactivity, with fear generalization. Fear generalization, measured via Linear Deviation Score (LDS), Mean Response across stimuli, and CS‐Differentiation, will be correlated with CAR to identify links between stress reactivity and fear learning. We will compare CAR between healthy adults and those with current or past mood disorders (anxiety or depression) and examine its relation to fear generalization. Lastly, we will use Pearson correlations to examine whether morning cortisol levels predict experiment‐induced HPA‐axis responses, using data from the follow‐up only as no similar probes were included in prior assessments.

#### 2.9.4 Epi(genetic) Analyses

Genetic variations in candidate genes and the polygenic risk scores for ANX will be derived from the recent GWAS of the PGC consortium (Strom et al. 2024). These will be applied individually as predictors of fear generalization in multiple logistic regression models on parameters calculated from the experiment.

DNAm values in genes related to HPA‐system (CRH, CRHR1, PACAP, ADCYAP1, SGK1, NR3C1) will be correlated with fear generalization, childhood adversity and life events by linear regression analyses. EWAS analyses on fear generalization index will be applied in a subset of samples with complete fear generalization and environmental information over the different recruitment waves. Multiple logistic regression modelling will be used to determine the contribution of DNAm to fear generalization parameters and the above‐mentioned environmental factors.

## 3 Discussion

Fear generalization may serve as a transdiagnostic marker of exaggerated defensive responses to stimuli resembling an initial threat condition, but evidence on its stability and role as a risk factor for anxiety is limited. Therefore, the current study aims to clarify several research questions and close these gaps, examining *N* = 226 children (third assessment) and *N* = 357 adults (second assessment) at follow‐up.

Our first question examines the long‐term stability of fear acquisition and generalization. Previous studies have demonstrated fair stability over relatively short to medium time intervals, ranging from 2 weeks to 8 months. Extending this work, our study investigates stability across a substantially longer period of three to 12 years. Given the broad age range, this design allows us to test whether fear acquisition and generalization reflect trait‐like mechanisms. Stable effects would support their role as trait‐like markers and risk factors for pathological anxiety, whereas instability would challenge this assumption. In the case of instability, an important next step would be to identify potential sources of this variability in fear learning, for example, influences of mental health status or dimensional anxiety symptoms. Parallel changes would indicate a state‐like function, suggesting fear generalization reflects dynamic symptom processes rather than a stable predisposition. Conversely, if stability is observed, this would support the interpretation of fear learning as a predictor of anxiety.

The second question addresses predicting later anxiety from fear learning, supplemented by psychometric, life event, (epi)genetic, and stress reactivity data. How molecular and environmental factors shape anxiety is complex, requiring integration of many variables. We will integrate polygenic risk score to capture the genetic liability and to test specific candidate genes for their influence on anxiety levels. By adding targeted DNAm data and environmental factors (e.g., childhood trauma and life events) between the recruitment waves, we construct a comprehensive multilevel model of how fear learning, molecular and environmental factors interact in anxiety. This approach captures the complexity of these processes within the organism. The inclusion of healthy participants allows us to test whether early fear generalization predicts later anxiety outcomes, distinguishing between predictive, non‐predictive, or unsystematic associations with subsequent symptom development. In doing so, this analysis evaluates the extent to which fear generalization functions as a risk marker for anxiety.

The third research question examines links between fear acquisition and generalization and HPA axis activity. HPA‐axis dysregulation is linked to stress‐related disorders including ANX, making these interactions important. Connecting stress responses to fear processes may reveal biological mechanisms underlying anxiety vulnerability and resilience.

### 3.1 Limitations

Repeated fear conditioning and generalization assessments may reduce fear and physiological arousal due to habituation to the threat stimulus. While a 3–12‐year interval likely minimizes this effect in adults, adolescents undergo a third measurement with the same paradigm, raising the risk of repeated measures bias. Although we check for habituation by asking which threat stimulus appeared in the last assessment, potential bias cannot be fully excluded. Yet, using different stimulus sets can impact the stability of fear acquisition across assessments, although generalization processes appear unaffected (Torrents‐Rodas et al. 2014). A further limitation concerns the interpretation of fear generalization as a stable vulnerability marker. Although the present study helps clarify the distinction between trait‐like and state‐like processes, it does not allow a definitive separation of these influences whether fear generalization reflects a stable predisposition or varies as a function of current symptom status. Since initial recruitment targeted psychiatrically healthy individuals, few may have developed pathological anxiety, limiting generalizability of fear generalization as a mechanism for ANX and predictor for pathological anxiety in clinical samples. A further issue relates to the heterogeneity introduced by variability in follow‐up intervals and developmental stages across the sample. In the adult cohort, follow‐up intervals vary substantially (3–12 years), resulting in differences in participants' age at reassessment as well as in their exposure to life events and risk periods for the onset of mental disorders. This variability may complicate the interpretation of longitudinal changes. In the child and adolescent cohort, although follow‐up intervals are more consistent, heterogeneity arises from variability in developmental stages due to differences in age at study entry. As developmental trajectories do not progress uniformly with chronological age, observed changes in fear learning may reflect both developmental processes and individual differences in maturation. Future studies incorporating more fine‐grained assessments of developmental stage may help to disentangle these effects.

### 3.2 Conclusion

In summary, this study investigates the stability of fear generalization as a trait marker and its role in anxiety development over a unique 3–12‐year interval. By combining fear learning mechanisms with psychometric, life event, molecular, and HPA axis data, we aim to advance understanding of anxiety development and inform targeted prevention, intervention, and treatment strategies.

## Author Contributions

**Jessica V. Reinhart:** data curation (equal), investigation (lead), project administration (lead), software (supporting), visualization (lead), writing – original draft preparation (lead). **Katharina Hutterer:** conceptualization (supporting), data curation (equal), investigation (supporting), project administration (supporting), software (lead), validation (equal), writing – review and editing (equal). **Heike Weber:** data curation (equal), resources (equal), writing – review and editing (equal). **Helena Strehl:** investigation (supporting), writing – review and editing (equal). **Miriam A. Schiele:** validation (equal), writing – review and editing (equal). **Katharina Domschke:** writing – review and editing (equal). **Andreas Reif:** writing – review and editing (equal). **Paul Pauli:** writing – review and editing (equal). **Ulrike Lueken:** writing – review and editing (equal). **Udo Dannlowski:** writing – review and editing (equal). **Tina B. Lonsdorf:** writing – review and editing (equal). **Bertram Müller–Myhsok:** data curation (equal), methodology (equal). **Jürgen Deckert:** conceptualization (equal), supervision (equal), resources (equal). **Marcel Romanos:** conceptualization (equal), supervision (equal), resources (equal). **Julia Reinhard:** conceptualization (equal), funding acquisition (equal), supervision (equal). **Angelika Erhardt–Lehmann:** conceptualization (equal), funding acquisition (equal), methodology (equal), supervision (equal), writing – original draft preparation (supporting), writing – review and editing (equal). **Andre Pittig:** conceptualization (equal), funding acquisition (equal), methodology (equal), resources (equal), software (supporting), supervision (equal), writing – review and editing (equal).

## Funding

The previous data collection and analysis was supported by a grant from the Deutsche Forschungsgemeinschaft (DFG; SFB‐TRR58, project Z02/2 and Z02/3 to J.D., K.D., A.R., P.P., U.L., U.D., T.L. and M.R.). The current study is supported by a grant from the Deutsche Forschungsgemeinschaft (DFG to J.R., A.P. and A.E., PI1269/5‐1 project number 499262975).

## Ethics Statement

The study was approved by the ethical committee of the medical board of the university of Würzburg (256/21) and conducted according to the ethical principles of the Helsinki Declaration.

## Consent

Written informed consent was obtained from all participants. For underage participants, additional consent was obtained from their parents or legal guardians.

## Conflicts of Interest

The authors declare no conflicts of interest.

## Permission to Reproduce Material From Other Sources

The authors have nothing to report.

## Data Availability

Data sharing is not applicable as no new datasets were analyzed. The data from earlier waves and analyses (A‐T1/1 or/2 and C‐T1‐T2) cited in the text are available per the respective journals' policies.
